# Small intestinal bacterial overgrowth and metabolic dysfunction-associated steatotic liver disease

**DOI:** 10.3389/fnut.2024.1502151

**Published:** 2024-12-17

**Authors:** Ziteng Wang, Wentao Tan, Jiali Huang, Qian Li, Jing Wang, Hui Su, Chunmei Guo, Hong Liu

**Affiliations:** ^1^Department of Gastroenterology, Beijing Shijitan Hospital, Capital Medical University, Beijing, China; ^2^Department of Gastroenterology, Beijing Ditan Hospital, Capital Medical University, Beijing, China

**Keywords:** metabolic dysfunction associated steatotic liver disease, metabolic dysfunction-associated steatohepatitis, small intestinal bacteria, breath test, hydrogen methane breath test

## Abstract

Small intestinal bacterial overgrowth (SIBO), characterized by alterations in both the type and quantity of bacteria in the small intestine, leads to impaired intestinal digestion and absorption that can cause a range of clinical symptoms. Recent studies have identified significant changes in the composition of the small intestinal microbiota and metabolomic profiles of patients with metabolic dysfunction-associated steatotic liver disease (MASLD). This study systematically reviewed and synthesized the available data to explore the association between SIBO and MASLD. Comprehensive literature searches of the Embase, PubMed, Web of Science, Ovid, and Cochrane databases were conducted. Article quality screening was performed using the Newcastle-Ottawa Quality Assessment Scale. Cross-sectional, cohort, and case–control studies were included. A total of 7,200 articles were initially screened, of which 14 were ultimately included for analysis. Individuals with SIBO in both the MASLD and non-MASLD groups were extracted and a chi-square test was performed to calculate the odds ratio (OR) and 95% confidence interval (CI). The I^2^ index was used to measure heterogeneity. For heterogeneity >50%, a random effects model was used. There was a clear association between SIBO and MASLD (OR = 3.09; 95% CI 2.09–4.59, *I*^2^ = 66%, *p* < 0.0001). Subgroup analyses by MASLD stage showed that the probability of SIBO positivity increased with MASLD lesion severity. After stratifying by the diagnostic methods for SIBO and MASLD, the meta-analysis results suggest a reduction in inter-group heterogeneity. For the MASLD subgroup diagnosed via liver biopsy, the OR was 4.89. A subgroup analysis of four studies that included intestinal permeability testing revealed an OR of 3.86 (95% CI: 1.80–8.28, *I*^2^ = 9%, *p* = 0.0005). A meta-regression analyses revealed that both race and regional development level significantly influenced the relationship between SIBO and MASLD (*p* = 0.010, *p* = 0.047). In conclusion, this meta-analyses provides strong evidence that SIBO may contribute to the development and progression of MASLD. The strongest associations were observed between lactulose breath testing, gut microbiota culture, liver biopsy diagnosis of MASLD, and SIBO detected through intestinal permeability testing. The primary sources of heterogeneity are race and developed regions.

**Systematic Review Registration:**

https://www.crd.york.ac.uk/PROSPERO/display_record.php?RecordID=427040.

## 1 Introduction

Non-alcoholic fatty liver disease (NAFLD) was first identified in 1980 and is estimated to affect 25–30% of the population in developed countries (1). NAFLD encompasses a spectrum of diseases, including simple fatty liver (SFL), nonalcoholic steatohepatitis (NASH), and cirrhosis (2, 3). In 2020, the European Conference redefined NAFLD as metabolic dysfunction-associated fatty liver disease (MAFLD), highlighting its close correlation with metabolism (4). In 2023, the Delphi Conference redefined it as metabolic dysfunction-associated steatotic liver disease (MASLD) (5). The primary pathological feature of MASLD is excessive fat deposition in liver cells. While primary MASLD is classified as an acquired metabolic stress-induced liver injury closely linked to insulin resistance and genetic susceptibility, secondary MASLD is caused by specific genetic factors (6, 7). Recent studies indicate that MASLD has a bidirectional relationship with various metabolic disorders, including elevated blood pressure, hyperuricemia, and obesity (8–10). The gut microbiota also plays a significant role in the occurrence and development of MASLD.

The adult gastrointestinal tract hosts the largest microbiome in the human body (11), consisting of diverse bacterial species that reproduce rapidly and maintain a stable composition across different gut regions, forming a complex ecosystem (12). Intestinal microbes help to regulate human metabolism and essential physiological processes such as digestion and immunity (13). An imbalance of the normal gut microbiota is implicated in major diseases such as obesity, diabetes, and colorectal cancer (14). Normally, the small intestine maintains a low concentration and diversity of bacteria (15, 16). Small Intestinal Bacterial Overgrowth (SIBO) is characterized by an abnormal increase in the quantity or diversity of bacteria in the small intestine. Clinical manifestations of SIBO are often nonspecific and are primarily characterized by excessive gas accumulation that is associated with abdominal distension, pain, constipation, diarrhea, weight loss, and progressive malnutrition (17–19). Risk factors for SIBO include anatomical abnormalities of the gastrointestinal tract, hypochlorhydria, intestinal motility disorders, age, certain medications, and conditions such as irritable bowel syndrome, celiac disease, and ulcerative colitis. Reliable SIBO diagnosis involves culturing small intestine aspirates during endoscopy. This enables the precise quantification of small intestinal bacteria. The diagnostic criterion for SIBO was initially a bacterial count >10^5^ colony-forming units (CFU) per milliliter of aspirate from the third portion of the duodenum but has been reduced to a cutoff of 10^3^ CFU/mL of aspirate (20–22). The lactulose hydrogen breath test (LHBT) and glucose hydrogen breath test (GHBT) are widely recognized as non-invasive tests for diagnosing SIBO with a sensitivity and specificity of 52.4/62.5% and 85.7/81.8%, respectively (23).

Recent studies suggest a correlation between gut microbiota, bacterial translocation, and hepatic steatosis (3). The gut microbiome influences host metabolism by secreting bioactive metabolites that impact the immune system and mucosal barrier permeability. Increased intestinal permeability from SIBO can facilitate the entry of microbiota metabolites and pathogenic factors into the human body (24). These substances circulate in the bloodstream and are transported to the liver via the portal vein. Human studies indicate that MASLD and SIBO patients exhibit elevated endotoxin levels. Endotoxin activates the pro-inflammatory cascade, thereby promoting the progression of MASLD (25). As intestinal barrier integrity is further compromised, dysfunction is exacerbated, further contributing to the development of SIBO. SIBO also affects MASLD by limiting the production of adipokines, particularly fasting-induced adipocyte factor (Fiaf), in adipose tissue. This inhibits the activity of lipoprotein lipase (LPL) which is important for fatty acid uptake and triglyceride deposition in hepatic adipocytes (26). Thus, SIBO may be a contributory factor in the pathogenesis of MASLD. The current study is a systematic review and meta-analysis of existing literature on SIBO and MASLD. The aim was to evaluate the association between SIBO and MASLD, summarize findings, and provide clinical data to guide future research.

Previous meta-analyses (18) have demonstrated strengths in research methodology and data integration concerning NAFLD and SIBO. These analyses systematically synthesized data from multiple independent studies, offering quantitative effect estimates like risk ratios or standardized mean differences to measure the impact of influencing factors on research outcomes. However, these analyses often did not explore the consistency and heterogeneity among studies. There is ongoing debate about the impact of acid suppressants on SIBO. Proton pump inhibitors (PPIs) and histamine-2 receptor blockers (H2RAs) are thought to increase the risk of SIBO (27). Thus, the current study included individuals using acid-suppressing agents, treating this as a covariate in the meta-regression analysis. Some prior studies also failed to use appropriate statistical techniques or conduct essential sensitivity analyses, compromising the rigor and reliability of the research findings. To address this, the present study used various diagnostic methods and conducted subgroup analyses to determine whether differences in diagnostic techniques would influence the outcomes. Lastly, previous meta-analyses may not have incorporated the most recent findings, preventing a comprehensive assessment of current evidence. The definition of MASLD has evolved and comprehensive articles summarizing and analyzing the latest diagnostic criteria are notably absent. This study addresses these limitations by elucidating the correlation between gut microbiota and MASLD and offering a detailed and structured analysis.

## 2 Materials and methods

This study is a meta-analysis conducted in accordance with Preferred Reporting Items for Systematic Reviews and Meta-Analyses (PRISMA) guidelines. Ethical approval was not required as no original clinical raw data were used. The study protocol is registered with the International Prospective Register of Systematic Reviews (PROSPERO ID: CRD42023427040).

### 2.1 Inclusion criteria

The types of research articles included in this study were observational, cohort, and case–control studies. Three independent authors (WZT, TWT and HJL) conducted searches in PubMed, Web of Science, Embase, Ovid Medline, and the Cochrane Library clinical trial databases. The searches focused on studies assessing the prevalence of SIBO in MASLD (case) and non-MASLD (control) groups and the number of participants in each group was extracted. Details of the databases and search strings are provided in Supplementary Appendices 1–5.

According to previous guidelines (1), the diagnosis of non-alcoholic fatty liver disease (NAFLD) can be confirmed through ultrasound examination, imaging studies, or biopsy, requiring a liver fat deposition of ≥5% and excluding other known liver diseases, including excessive alcohol consumption. In 2020, Europe introduced MAFLD and redefined fatty liver (13). While the criteria for diagnosing fat deposition remained the same, metabolic dysfunction was prioritized. Driving factors for metabolic risk include type 2 diabetes and overweight/obesity classified by race-specific body mass index (BMI). Diagnosis can also be made for patients having at least two of the following risk factors: waist circumference, blood pressure, plasma triglycerides, plasma high-density lipoprotein cholesterol, prediabetes, insulin resistance determined by homeostasis model assessment, and plasma high-sensitivity C-reactive protein. In 2023, the Delphi consensus conference jointly proposed a new nomenclature, MASLD that emphasizes the role of metabolic cardiovascular risk factors in NAFLD pathogenesis. For fatty liver disease patients with a normal BMI and no diabetes, MASLD diagnosis requires the presence of at least one metabolic cardiovascular risk factor, including obesity or overweight, type 2 diabetes (T2DM), hypertension, reduced high-density lipoprotein cholesterol levels, and elevated plasma triglyceride levels. Thus, articles were re-screened based on the latest standards.

SIBO is diagnosed using a breath test or biopsy. Breath test diagnosis involves a hydrogen, methane, or mixed hydrogen and methane test and an oral solution composed of glucose or lactulose. Biopsy diagnosis requires the culture of duodenal and jejunal aspirates.

Populations with viral hepatitis, alcoholic cirrhosis, and interventions that impact the treatment of MASLD or small intestinal bacterial overgrowth, including gastrointestinal system-related surgery, opioids, irritable bowel syndrome, intestinal motility disorder, small bowel diverticulum, systemic sclerosis, and hypothyroidism, were excluded from the analyses. This study had no geographic restrictions.

### 2.2 Study selection process

The databases were screened according to the publication title. The included publications were further screened by abstract content. It was then verified that the full text met the inclusion criteria. Three independent authors resolved any inconsistencies through consultation.

### 2.3 Data extraction data

Table 1 systematically summarizes the data in each study including the study title, first author name, year of publication, country where the study was conducted, participant demographics, and MASLD and SIBO diagnostic methods. The relative risks (RR), odds ratios (OR), 95% confidence intervals (CI), and *p* values were extracted from the original article. If these data were absent in the original text, the number of people in each group was extracted, a chi-square test was used to calculate the OR and related values, and numeric conversions were performed. Patient age, gender, BMI, developed country status, and use of gastric acid inhibitors were also collected. The raw data were analyzed, then use these calculated data for subgroup analysis.

**Table 1:** Observational studies on the association between small intestinal bacteria overgrowth and metabolic dysfunction associated steatotic liver disease.

No	First author	Country Year of publication	Type of the Study	Diagnostic criteria of SIBO	Diagnostic criterion of MASLD	Sex	Age (year)	BMI (kg/m2)/ weight (kg)	Intestinal permeability test	Newcastle Ottawa Scale
1	Sabaté et al. (31)	France 2008	Case–control	Glucose hydrogen breath test	Liver biopsy	17 male	40.7 ± 11.4	46.1 ± 6.4	No data	7
2	Miele et al. (29)	Italy 2009	Case–control	Glucose breath testing (gbt)	Liver biopsy	Volunteers: 22male NAFLD: 30male	Volunteers: 28–45 NAFLD: 32–54	Volunteers: 23.85–25.22 NAFLD: 24.39–27.97	Chromium-51 Ethylenediamine Tetraacetate Excretion Testing Immunohistochemical Studies of Duodenal Zonula Occludens-1 Expression	7
3	Nier et al. (30)	Germany 2017	Case–control	Glucose h2 breath test	Ultrasound	Controls: 15male	Controls: 6.6–8.1	Controls: 16.2–17.8	The levels of soluble CD14 D-lactate plasminogen activator inhibitor-1 activity Lipopolysaccharide-binding protein interleukin-6	8
						NAFLD: 8 male	NAFLD: 6.9–9.0	NAFLD: 19.4–24.2		
4	Gkolfakis et al. (35)	Greece 2023	Cross-sectional	Duodenal fluid from the 3rd–4th part of duodenum	Histological or biochemical or radiological diagnosis	52 male	54 ± 11.9	88.3 ± 19.6	No data	8
5	Shanab et al. (32)	Ireland 2010	Case–control	The lactulose breath hydrogen test (LHBT)	Liver biopsy	Controls: 7 male	Controls: 50.80 ± 2.4	Controls: 26.25 ± 0.9340	Plasma lipopolysaccharide-binding protein Expression of Toll-like receptor 2 Toll-like receptor 4 on CD14-positive cells pro-inflammatory cytokines	6
						NASH: 8 male	NASH: 51.17 ± 2.4	NASH: 30.00 ± 0.7940		
6	Fitriakusumah et al. (36)	Indonesia 2019	Cross-sectional	Glucose hydrogen breath test (GHBT)	Transabdominal ultrasound examination	52 male	58 (22–78)	76.6% Obesity	No data	8
7	Fialho et al. (25)	USA 2016	Case–control	The glucose H2/CH4 breath test	Abdominal imaging examination	92 male	59.76 ± 0.77	13 Underweight	No data	7
								148 Normal weight		
								98 over weight		
								113 Obese		
8	Belei et al. (6)	Romania 2017	Case–control	Glucose hydrogen breath test (GHBT)	Abdominal imaging	Overweight and SIBO positive: 29 boys	Overweight and SIBO positive: 15.52 ± 2.43	Overweight and SIBO positive: 27.92 ± 3.08	No data	6
						Overweight and SIBO negative: 51 boys	Overweight and SIBO negative: 14.18 ± 2.17	Overweight and SIBO negative: 27.35 ± 3.12		
						Control group: 79 boys	Controls: 15.28 ± 2.25	Controls: 20.53 ± 2.15		
9	Ortiz Lopez et al. (38)	Chile 2024	Cross-sectional	Lactulose breath tests	Liver biopsy	Control: 14 female	Control: 38.5	Control: 37.9	No data	8
						MASL: 14 female	MASL: 34.3	MASL: 47		
						MASH-HF: 16 female	MASH-HF: 37.7	MASH-HF: 44.5		
						MASH-F: 18 female	MASH-F: 42.9	MASH-F: 41.8		
10	Troisi et al. (39)	Italy 2017	Cohort study	Lactulose hydrogen breath test	Ultrasonography	Control: 9 male	Control: 11.25 ± 2.26	Control: 17.27 ± 2.09	The high-performance liquid chromatography of lactulose and mannitol in urine.	7
						All Obese: 13 male	All Obese: 11.55 ± 2.12	All Obese: 27.63 ± 4.56		
11	De Oliveira et al. (11)	Brazil 2020	Cross-sectional	Lactulose H_2_/CH_4_ breath test	Liver biopsy	With metabolic syndrome: 5 female	With metabolic syndrome: 47.5 ± 10.5	With metabolic syndrome: 29.9 ± 3.46	No data	7
						Without metabolic syndrome: 13 female	Without metabolic syndrome: 49.5 ± 9.8	Without metabolic syndrome: 33.8 ± 5.8		
12	Stepanov et al. (34)	Spain 2019	Case–control	Lactulose breath test (LBT)	Liver biopsy or imaging or biochemical studies	Obese individuals with SIBO: 18 (60.0%) male	Obese individuals with SIBO: 11.77 ± 2.65	Obese individuals with SIBO: 24.86 ± 3.72	No data	6
						Obese children without SIBO: 17 (60.7%) male	Obese children without SIBO: 10.82 ± 2.80	Obese children without SIBO: 24.40 ± 3.81		
						Control: 18 (53.3%) male	Control: 11.80 ± 2.48	Control: 17.06 ± 0.84		
13	Shi et al. (33)	China 2021	Case–control	Lactulose hydrogen breath test	Imaging examination	MAFLD: 54 male	MAFLD: 48.52 ± 12.34	SIBO positive: 26.52 ± 2.27	No data	6
						control: 26 male	control: 46.39 ± 8.89	SIBO negative: 26.13 ± 2.45		
14	Mikolasevic et al. (37)	Croatia 2021	Cross-sectional study	Aspiration of the descending duodenum	FibroScan and liver biopsy	56 (47.9%) male	58.3 ± 11.7	33.4 ± 5.3	No data	8

### 2.4 Statistical analysis

The comprehensive meta-analysis software, Revman (Review Manager version 5.1, Copenhagen: Nordic Cochrane Centre, Cochrane Collaboration, 2011), was used for this study. Data were extracted from each article, the OR and 95% CI were calculated, and a meta-analysis was conducted. Heterogeneity was assessed using the I2 index, where values of 0–25% indicate no significant heterogeneity, 26–50% indicate low heterogeneity, 51–75% indicate moderate heterogeneity, and > 75% represent high heterogeneity. Fixed effects models were used for heterogeneity <50%, while random effects models were used for heterogeneity ≥50%. Sensitivity analysis involved systematically excluding one study at a time and generating sensitivity plots using Stata (StataCorp version 18.0, LLC4905 Lakeway Drive College Station, TX 77845, USA). Stratified analyses of SIBO prevalence by MASLD stage were conducted, as well as differences in the SIBO and MASLD diagnostic methods To identify sources of heterogeneity, factors such as age, gender, body mass index (BMI), use of gastric acid suppressants, country development status, and study type were classified, and meta-regression analysis was performed using Stata (StataCorp version 18.0, LLC4905 Lakeway Drive College Station, TX 77845, USA).

### 2.5 Bias risk

Article quality was assessed by two independent investigators (WZT and TWT) using the Newcastle-Ottawa Quality Assessment Scale (NOS). Different criteria were used for each study type. For cohort studies, NOS assessment accounts for the representativeness of the exposed cohort, whether the selection method of the non-exposed cohort is correct, whether the identification of exposure factors is reasonable, and whether there is a need to observe at the beginning of the study. The comparability of the exposed and unexposed groups should be considered when determining the outcome indicators, design, and statistical analysis, whether the evaluation of the research results is sufficient, whether follow-up is long enough, and whether follow-up of the exposed and non-exposed groups is sufficient. For case–control studies, NOS assessment considers whether the case identification is appropriate, whether the selected cases are representative, whether the selection and identification of the control groups are reasonable, whether the comparability of cases and controls is considered in the design and statistical analysis, and whether the identification of exposure factors is accurate, whether the same method was used to identify exposure factors for the cases and controls, and whether the non-response rates were the same for the cases and controls. The maximum NOS score is 9 points, with 6–8 points considered good research quality (28, 68).

## 3 Results

**Figure 1:**
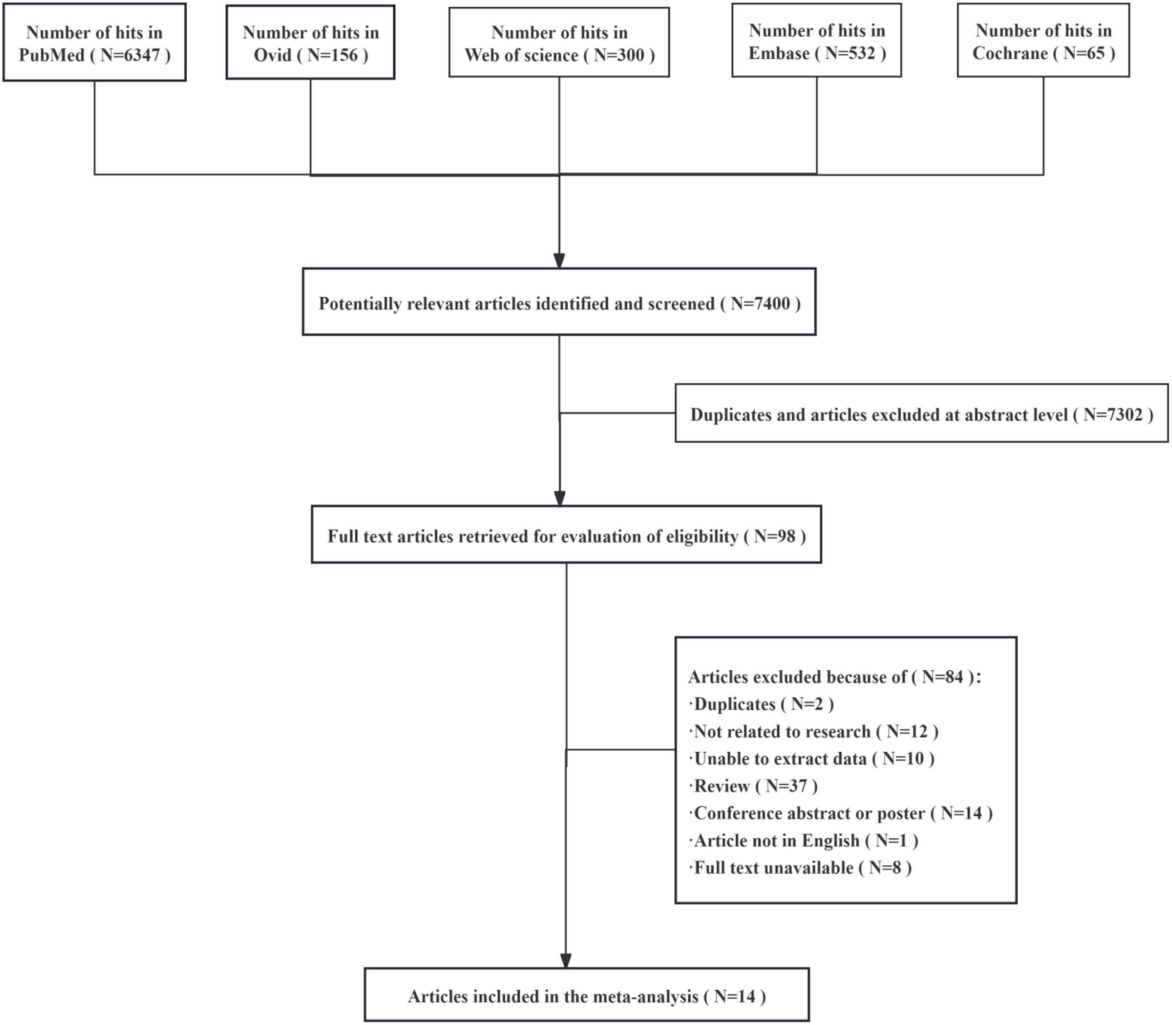
Flow diagram of the studies screened and included in the meta-analysis.

The initial search yielded 7,400 matches, of which 7,302 studies were excluded after further screening and the removal of duplicates and articles with titles that did not match the topic. A total of 98 full-text articles were included in the final screening, of which 84 were excluded because they had duplicate records (*N* = 2), unrelated research (*N* = 12), data that could not be extracted (*N* = 10), were not in English (*N* = 1), had unavailable full text (*N* = 8), or were overview studies (*N* = 37) or meeting summaries or posters (*N* = 14) (Figure 1). After exclusion, 14 studies were included in the final analysis.

### 3.1 Study patients and characteristics

This meta-analysis was conducted on eight case–control studies (6, 25, 29–34), five cross-sectional studies (11, 35–38) and one cohort study (10) that included populations from 14 different countries (Table 1). All studies included patients with various types of MASLD that ranged from 0 to 80 years of age. Liver disease was diagnosed by liver biopsy in six of the studies (11, 29, 31, 32, 37, 38). SIBO was diagnosed by lactulose (34, 38), lactulose hydrogen (32, 33, 39), lactulose hydrogen and methane (11), glucose (29), glucose hydrogen (6, 30, 31, 36), glucose hydrogen and methane (25), or duodenal aspirate quantitative culture (35, 37). Four articles included intestinal permeability testing (29, 30, 32, 39).

### 3.2 Association between SIBO and MASLD risk

**Figure 2:**
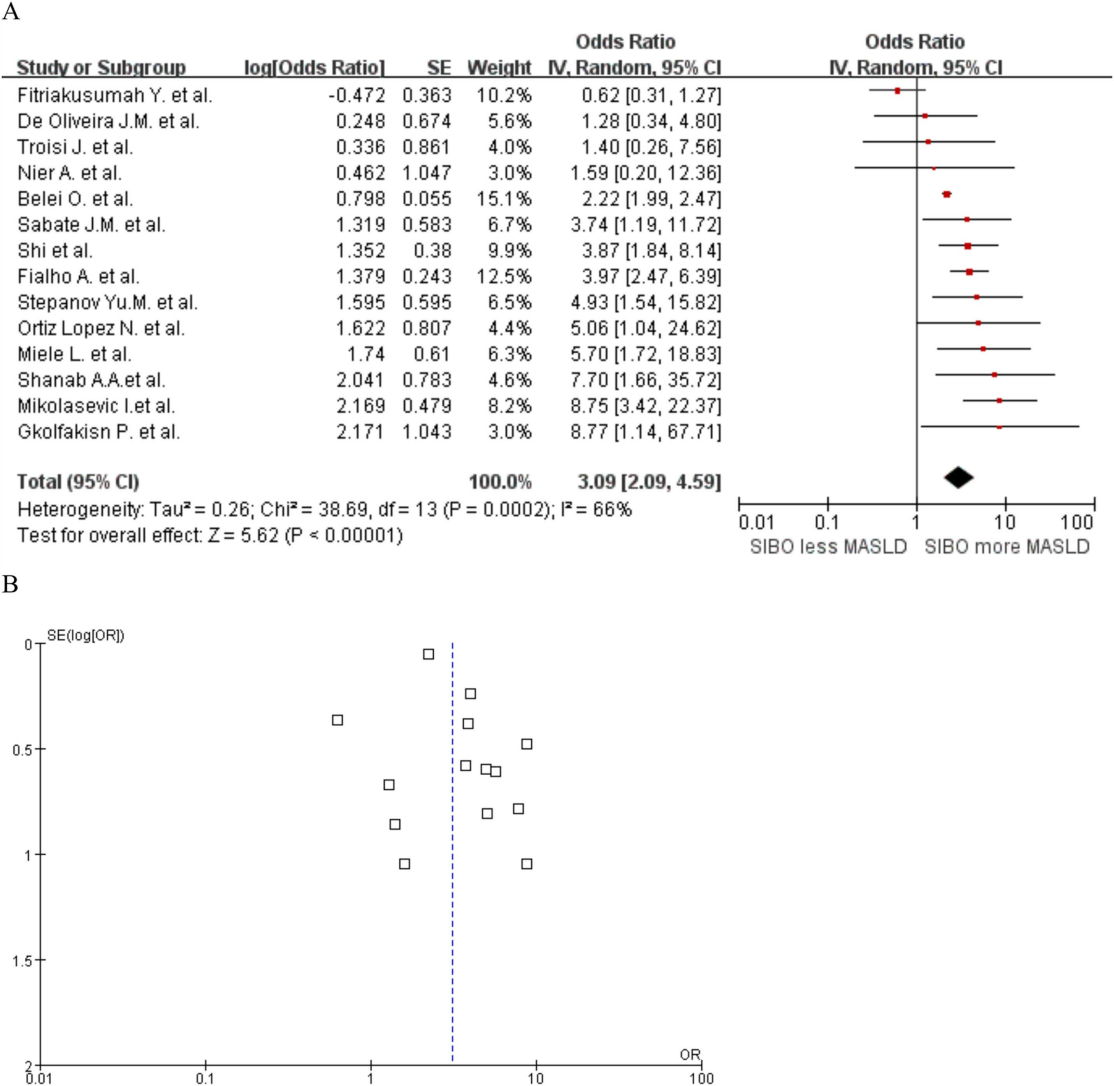
**(A)** Forest plot of the meta-analysis of SIBO and MASLD. OR, odds ratio; CI, confidence interval. **(B)** Funnel plot to detect potential publication bias.

**Figure 3:**
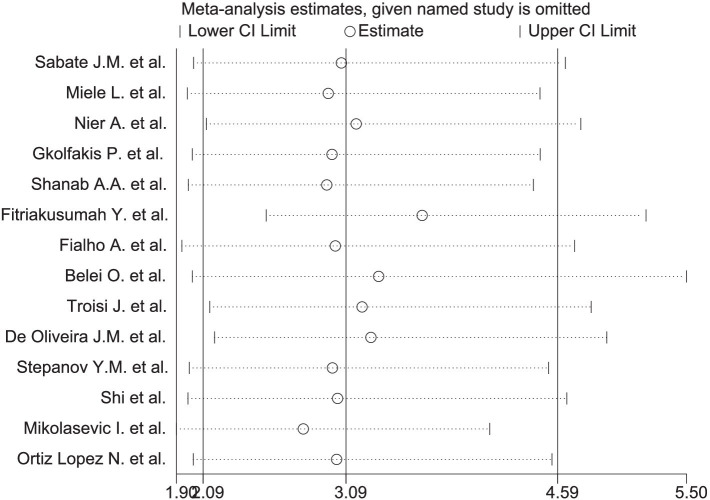
Sensitivity analysis of SIBO and MASLD.

A significant correlation was observed between MASLD and SIBO, with a combined OR of 3.09 (95% CI 2.09–4.59, *I*^2^ = 66%, *p* < 0.0001) (Figure 2A). Since the heterogeneity between studies was high, a random-effects model was used. A sensitivity analysis was conducted on the 14 studies (Figure 3). The sensitivity analysis results indicate that the results are stable.

**Figure 4:**
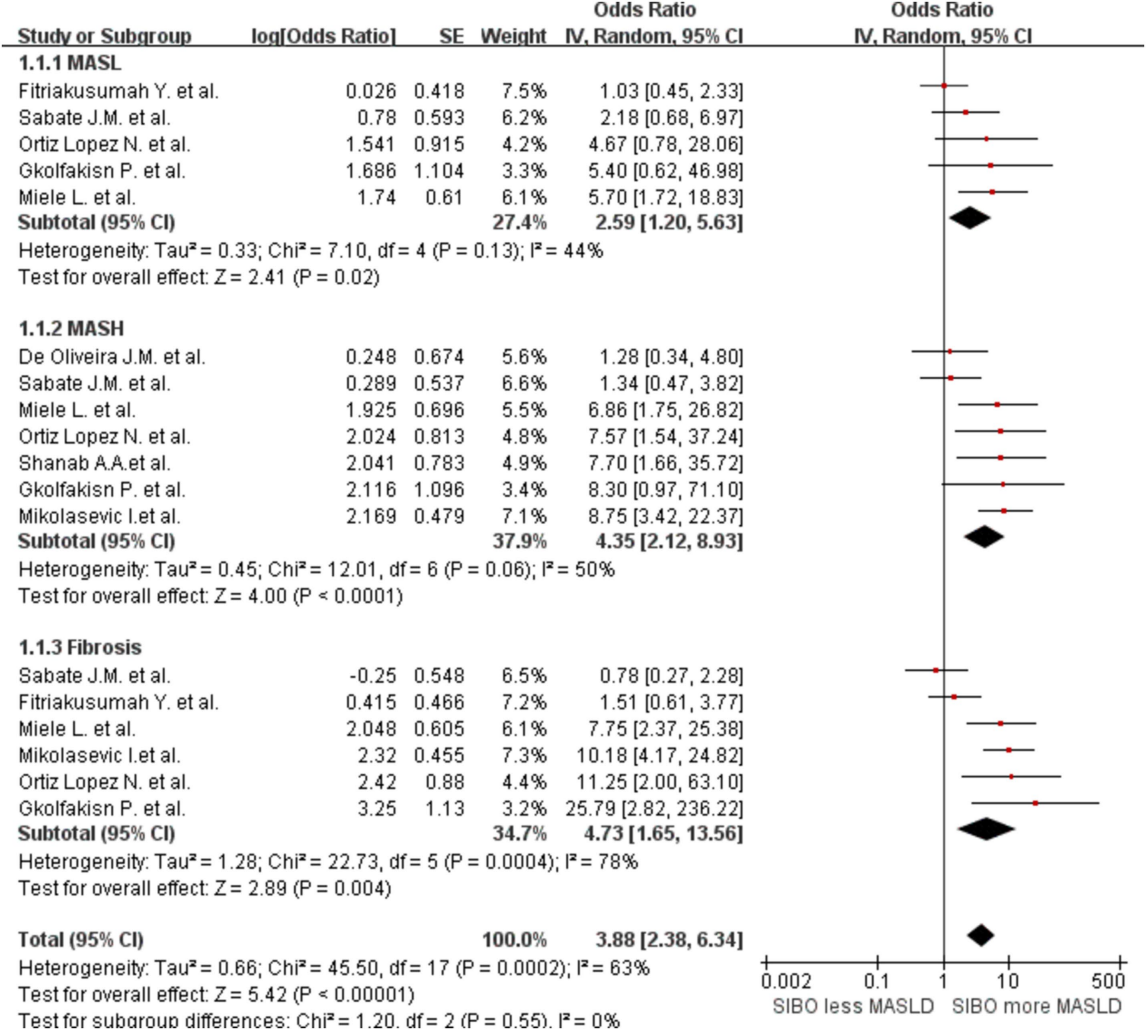
Subgroup analyses by stage of MASLD progression. A forest plot was created to illustrate the association between SIBO and MASLD. OR, odds ratio; CI, confidence interval.

To further explore the relationship between SIBO and MASLD at various stages of disease progression, we performed a stratified analysis. We classified MASLD patients into three stages based on clinical and pathological criteria. A subgroup analysis was then conducted by MASLD stage and the SIBO positivity rate was shown to increase gradually by MASL, MASH, and fibrosis stage. The heterogeneity between studies increased accordingly. The MASL group OR was 2.59 (95% CI 1.20–5.63, *I*^2^ = 44%, *p* = 0.02), and the MASH group OR was 4.35 (95% CI 2.12–8.93, *I*^2^ = 50%, *p* < 0.0001). The fibrosis group OR was 4.73 (95% CI 1.65–13.56, I^2^ = 78%, *p* = 0.004) (Figure 4).

**Figure 5:**
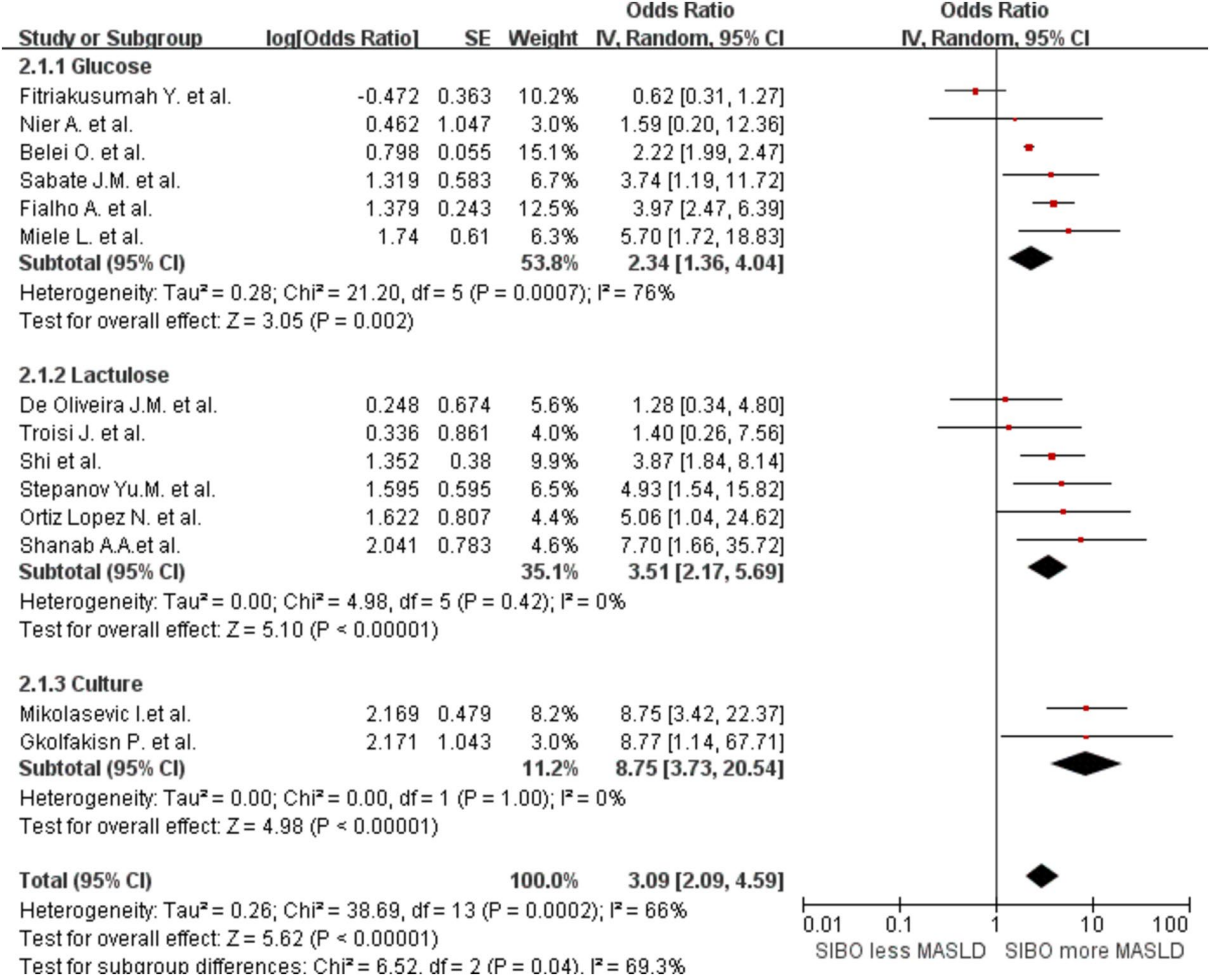
Subgroup analyses by diagnostic method of SIBO. A forest plot was created to illustrate the association between SIBO and MASLD. OR, odds ratio; CI, confidence interval.

**Figure 6:**
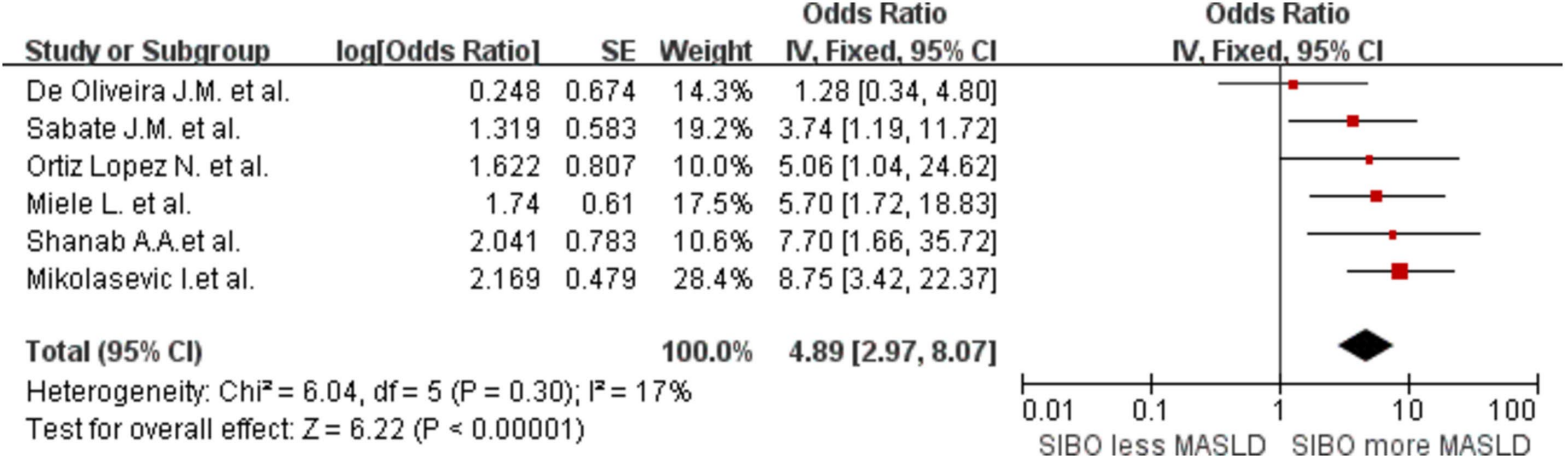
A forest plot of the meta-analysis of SIBO and MASLD patients diagnosed through liver biopsy. OR, odds ratio; CI, confidence interval.

In addition, to determine whether the diagnostic methods influence the outcomes, we conducted a comprehensive analysis of the diagnostic approaches for SIBO and MASLD, followed by a stratified assessment. We divided the data into three groups based on the diagnostic methods for SIBO: one group was categorized into the lactulose and glucose subgroups according to the type of substrate used in the breath test, while the other group consisted of the duodenal culture medium group. This grouping strategy allows for a more comprehensive analysis of how different substrate types and bacterial cultures influence the diagnostic outcomes of SIBO. The glucose group OR was 2.34 (95% CI 1.36–4.04, I^2^ = 76%, *p* = 0.002), and the lactulose group OR was 3.51 (95% CI 2.17–5.69, *I*^2^ = 0%, *p* < 0.00001). The Culture group OR was 8.75 (95% CI 3.73–20.54, *I*^2^ = 0%, *p* < 0.00001) (Figure 5). The results indicate that the heterogeneity within the lactulose group and the bacterial culture group has decreased compared to the before; however, some heterogeneity still remains. Then, the studies were also stratified by MASLD diagnostic method. A meta-analysis of MASLD subgroups diagnosed using liver biopsy had an OR of 4.89 (95% CI 2.97–8.07, I^2^ = 17%, *p* < 0.0001) (Figure 6). The heterogeneity between studies was significantly reduced.

**Figure 7:**
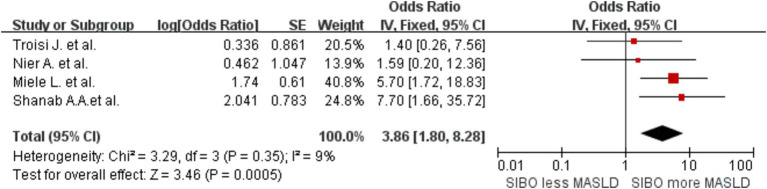
A forest plot of the meta-analysis of SIBO and MASLD patients who received intestinal permeability testing.

To clarify the mechanism linking SIBO and MASLD, particularly whether changes in intestinal permeability (i.e., “leaky gut”) influence the gut-liver axis, thereby altering fat metabolism and promoting the onset and progression of MASLD, we conducted a meta-analysis of relevant studies that include intestinal permeability testing. A meta-analysis of four studies had an OR of 3.86 (95% CI 1.80–8.28, *I*^2^ = 9%, *p* = 0.0005) (Figure 7). The heterogeneity was extremely low.

**Figure 8:**
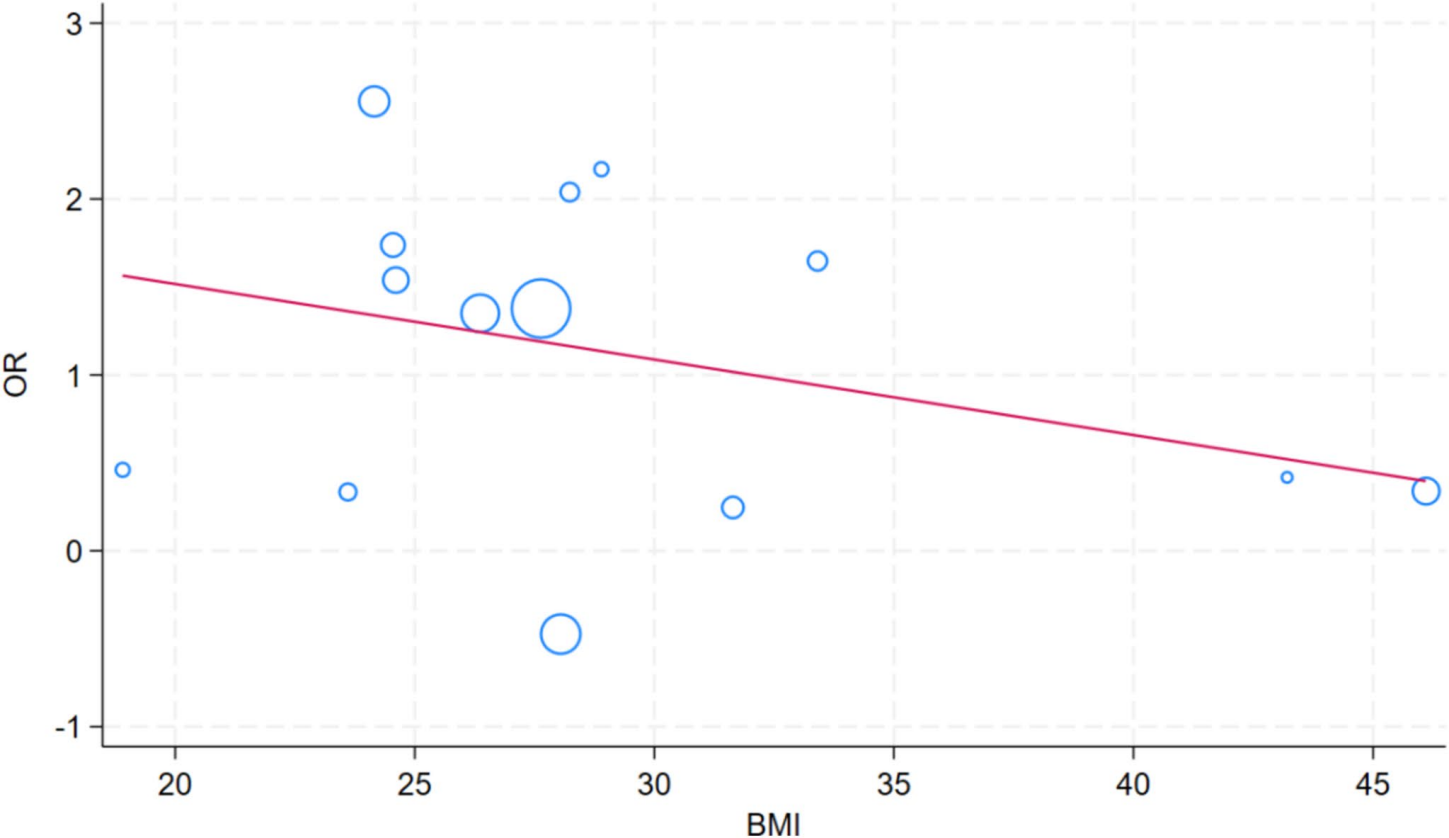
Bubble plot of meta-regression based on BMI.

To identify the sources of heterogeneity in the study, we included the following factors in the meta-regression analysis: age, sex, BMI, use of gastric acid suppressants, ethnicity, whether the study was conducted in a developed country, and the study design (e.g., cross-sectional studies, cohort studies, etc.). The aim was to assess the impact of these potential factors on the overall findings and to better understand how they contribute to variability across studies. A meta-regression analysis of factors that impact the SIBO and MASLD revealed significant differences by race (*p* = 0.010) and developed country (*p* = 0.047) (Table 2). The role of BMI in meta regression analysis is not significant (Figure 8).

**Table 2:** Multivariable meta-regression model for moderators of small intestinal bacteria overgrowth and metabolic dysfunction associated steatotic liver disease.

Moderator	Coefficient	S.E.	95% C.I. Low	95% C.I. High	*p*-value
BMI	−0.020	0.037	−0.093	0.052	0.582
Sex	1.348	1.294	−1.188	3.883	0.297
Use of PPI/H_2_	−0.731	1.253	−3.188	1.725	0.560
Age	0.027	0.020	−0.013	0.067	0.181
Whether developed country	1.728	0.868	0.026	3.429	**0.047**
Type of study	−0.165	0.593	−1.328	0.998	0.781
Race	−3.350	1.298	−5.894	−0.805	**0.010**

C.I., confidence interval; BMI, body mass index; SIBO, small intestinal bacterial overgrowth; PPI, proton pump inhibitor; H_2,_ H_2_-receptor antagonists.

Bold indicates statistically significant values.

### 3.3 Sensitivity analysis

A sensitivity analysis was conducted on the 14 studies using Stata (StataCorp version 18.0, LLC, 4905 Lakeway Drive, College Station, TX 77845, USA) (Figure 3). After excluding any one study, the combined results of the remaining 13 studies remained statistically significant and consistent with the original combined results.

### 3.4 Assessment of publication bias

To assess potential publication bias, funnel plots were constructed using the effectiveness estimation and accuracy of each study (Figure 2B). The plot was relatively symmetrical, but publication biases favoring positive results still existed. Further subgroup analyses of patients diagnosed with NAFLD by liver biopsy and of studies that used intestinal permeability testing were also conducted. Heterogeneity by MASLD stage was relatively low. Heterogeneity in MASLD patients diagnosed by liver biopsy and in studies including intestinal permeability testing was significantly lower, with no significant risk of publication bias.

## 4 Discussion

### 4.1 Main findings

The gut microbiome plays a significant role in regulating human health (34, 40, 41) by inducing systemic inflammation, thereby causing metabolic syndrome and other conditions. Several recent studies (42, 43)have documented alterations in the gut microbiota of patients with chronic liver disease and cirrhosis. Imbalances in the intestinal microbiome impact liver metabolism and hormone levels to varying extents. Of these, SIBO is most prevalent in patients with MASLD (39). The gut-liver axis, involving the dynamic interplay between gut microbiota and liver function, provides a theoretical basis for these observations (27, 31).

Under normal physiological conditions, the intestinal tract secretes immunoglobulin A (sIgA) which helps to maintain homeostasis in the small intestine (44). In patients with an imbalanced intestinal microbiome, excessive bacterial growth in the small intestine disrupts sIgA and the intestinal migratory myoelectric complex (MMC). This microbial overgrowth, including both aerobic and anaerobic organisms, is likely to accelerate growth through a positive feedback loop. This increases intestinal permeability, facilitating the entry of bacterial-derived compounds into the systemic circulation. These compounds interfere with intestinal absorption and deprive the body of nutrients, resulting in abdominal pain, diarrhea, and indigestion (45). The portal vein, which supplies blood to the liver, is rich in digested products from the gut, including those related to SIBO. The liver is first exposed to bacterial endotoxins, such as lipopolysaccharide (LPS) (41, 46), which activates TLR4 and CD14 receptors, inducing the production of pro-inflammatory cytokines, such as tumor necrosis factor-*α*, interleukin-1*β*, interleukin-6, and interleukin-8. The overproduction of these cytokines leads to chronic liver inflammation and insulin resistance. M1-polarized Kupffer cells and M1 macrophages stimulate the liver to produce triglycerides and activate hepatic stellate cells (HSCs) through the release of IL-1*β* (47, 48), further promoting insulin resistance and inflammation. This process may play a key role in the pathogenesis of MASLD, liver fibrosis, and liver cancer.

Our study confirms a significant correlation between SIBO and MASLD. However, due to the high level of inter-study heterogeneity, subgroup analyses were conducted by MASLD stage. As MASLD progresses, SIBO incidence was shown to gradually increase. SIBO may, in turn, exacerbate the inflammatory response, worsening the progression of MASLD. This finding aligns with previous studies showing that the prevalence of SIBO is higher in patients with more severe liver diseases (18). Our analysis indicates that when lactulose and bacterial culture methods are used to diagnose SIBO, the heterogeneity between groups is significantly lower compared to the initial analysis. However, some degree of heterogeneity remains. This phenomenon may be attributed to the relatively low sensitivity of glucose as a substrate for specific bacterial populations, which can affect diagnostic accuracy. In contrast, lactulose demonstrates higher sensitivity to a broader range of microbial communities, improving the accuracy of SIBO diagnosis (49). Furthermore, microbial culture (50), as a reliable detection method, can simulate and maintain the growth conditions of microorganisms *in vitro*, thereby providing a more accurate representation of the microbial community structure and function across different samples. Consequently, the use of microbial culture reduces inter-group heterogeneity significantly compared to other methods, reflecting a higher degree of consistency in SIBO diagnosis. Subgroup analysis based on liver diagnostic methods revealed a strong correlation between liver biopsy-based diagnosis of MASLD and SIBO, with low heterogeneity between groups. This can be attributed to the fact that liver biopsy is considered the gold standard for diagnosing MASLD, as it allows for direct sampling of liver tissue to evaluate pathological features such as fat accumulation, inflammation, and the degree of fibrosis. Compared to non-invasive methods like ultrasound, CT, or blood markers, liver biopsy provides more accurate histological information (51), particularly in assessing the grading and staging of liver injury. This greater diagnostic precision likely contributes to the stronger and more consistent association observed between SIBO and MASLD in studies using liver biopsy.

Stratification by intestinal permeability testing, which assesses the degree to which the intestinal wall allows the passage of liquids, nutrients, and microorganisms, was also conducted. This test is used to assess the health of the intestinal mucosa and determine whether intestinal barrier function is compromised. We include articles containing intestinal permeability testing in subgroup analysis, the findings indicated a robust correlation between SIBO and MASLD, with no significant heterogeneity across the included studies. Therefore, we hypothesize that “leaky gut” may disrupt the normal function of the gut-liver axis, leading to an increase in intestinal permeability (52). This dysfunction allows harmful substances, bacterial products, and endotoxins from the intestinal lumen to translocate into the bloodstream. These substances can then reach the liver via the portal vein, where they may trigger immune and inflammatory responses (53). This cascade of immune activation and inflammation can subsequently interfere with the liver’s lipid metabolism, promoting fat accumulation and metabolic dysregulation. As fat metabolism becomes dysregulated, the deposition of lipids in the liver intensifies, which may act as a key pathogenic factor in the initiation and progression of MASLD (54).

A meta-regression analysis was used to investigate factors affecting the relationship between SIBO and MASLD, including gender, age, use of acid suppressants, bmi, study area development status, race, and article type. Our research finding that racial differences represent an important factor contributing to the variation in the correlation between SIBO and MASLD. These differences are likely due to a combination of genetic susceptibility (55), environmental, and lifestyle factors. Genetically, different ethnic groups exhibit variations in the regulation of metabolic pathways, immune responses, and gut microbiota composition (56), which may influence their susceptibility to both SIBO and MASLD. Populations of European descent have been shown to exhibit greater sensitivity to insulin, lipid metabolism, and liver fat accumulation, making them more prone to developing MASLD (57). These genetic factors could make certain ethnic groups more responsive to dietary and environmental factors that influence fat metabolism, thereby increasing their risk of developing MASLD. Furthermore, ethnic differences in immune system functioning may also play a role in modulating both gut barrier integrity and the inflammatory responses that drive MASLD. In addition to genetic factors, differences in dietary habits and lifestyle also contribute significantly to the incidence of both SIBO and MASLD across racial groups. Diets high in fiber, plant-based foods, and refined sugars, may promote changes in gut microbiota that predispose individuals to SIBO. Diets rich in refined carbohydrates and fats, common in Western populations, can exacerbate gut dysbiosis and increase intestinal permeability (58), both of which are linked to an increased risk of SIBO and MASLD. Our research also found that whether a region is developed plays a crucial role in affecting SIBO and MASLD, with the dietary structure in different regions being a key factor. In developed regions, diets are typically high in refined carbohydrates and animal fats, which can contribute to an imbalance in the gut microbiota and promote the development of SIBO through various mechanisms. Refined carbohydrates, such as white bread, sugary snacks, and sweetened beverages, provide readily available energy for harmful bacteria in the small intestine (59), potentially leading to bacterial overgrowth. Additionally, high intake of animal fats may alter the gut microbiome (60), reducing the diversity of beneficial bacteria, and fostering an environment conducive to SIBO. At the same time, this dietary pattern is also a significant contributor to liver fat accumulation, which plays a central role in the development of MASLD. Excessive refined sugar and fat intake can lead to lipid deposition in the liver (61), increasing metabolic stress and promoting the onset of metabolic liver diseases. Thus, the dietary habits typical of developed regions contribute to the heightened risk of both SIBO and MASLD through their effects on the gut microbiota and liver metabolism.

Obesity plays a crucial role in the pathogenesis of SIBO and MASLD. The study by Ierardi et al. (62) demonstrated that obese individuals are at a higher risk of developing SIBO, which is often accompanied by an increased intake of carbohydrates and refined sugars, coupled with a lower intake of dietary fiber. Additionally, obesity may increase the risk of SIBO through mechanisms such as altered intestinal motility and gut dysbiosis, while simultaneously exacerbating MASLD through processes like fat accumulation and insulin resistance (63, 64). Although obesity has a significant impact on both SIBO and MASLD, the underlying mechanisms linking these three conditions remain complex and warrant further investigation. In our study, the relationship between BMI and both SIBO and MASLD was not found to be significant, which may be explained by several factors. First, the mechanisms through which obesity influences these two diseases appear to be relatively independent. While obesity contributes to the development of SIBO by altering intestinal motility and promoting dysbiosis, it exacerbates MASLD through fat accumulation, insulin resistance, and other metabolic disruptions. Although both SIBO and MASLD are linked to metabolic dysfunction and changes in the gut microbiota, their respective pathophysiological mechanisms are distinct, which may explain the lack of a direct significant association between obesity and these two conditions in our study.Second, gut microbiota dysbiosis may represent a common pathway through which obesity influences both SIBO and MASLD. Obesity is associated with a reduction in gut microbiota diversity (65), which could contribute to both the onset of SIBO and the progression of MASLD. Dysbiosis may alter gut barrier function and affect the secretion of metabolites, thereby promoting bacterial overgrowth in the small intestine and worsening liver steatosis (66, 67). Thus, gut microbiota dysbiosis may act as a potential mediator of obesity’s effects on these two diseases. Furthermore, the relationship between BMI and the risk of SIBO and MASLD may be non-linear. The impact of obesity on these conditions may exhibit a threshold effect, meaning that the influence of BMI on SIBO and MASLD may be more pronounced below certain BMI thresholds, but less significant in individuals with higher BMI. This non-linear relationship may not be adequately captured by linear regression models, which could explain the lack of significant findings in our analysis.Lastly, the evolving diagnostic criteria for MASLD may have influenced the results of our study. Given that the majority of our study population consisted of obese individuals, the generalizability of BMI effects across different BMI categories may be limited.In conclusion, while the direct association between BMI and SIBO and MASLD was not statistically significant in our study, obesity remains a key factor influencing both conditions through multiple biological pathways. Further research is needed to clarify the complex interplay between obesity, gut microbiota dysbiosis, and the development of these diseases.

### 4.2 Advantages of the meta-analysis

Previous study (48) has primarily investigated the relationship between SIBO and NAFLD in specific populations, such as children, and have confirmed the existence of an association between SIBO and NAFLD in this group. However, our study encompasses a broader age range, without any age restrictions. By including participants across the entire age spectrum, our research offers more generalizable findings, which can provide a more comprehensive understanding of the relationship between SIBO and NAFLD. This approach allows for more applicable conclusions that can inform clinical practice for patients of various age groups. Wijarnpreecha et al. (41) focused solely on the correlation between SIBO and NAFLD, while Gudan et al. (18) performed a stratified analysis of NAFLD across different stages of progression. The results from Gudan et al. revealed that the incidence of SIBO in NAFLD patients was 35%, with the highest incidence observed in NASH patients, reaching 41.1%. These findings are generally consistent with our results. However, compared to previous studies, our research offers a more in-depth and comprehensive analysis. In addition to stratifying MASLD patients by disease stage, we expanded our investigation by examining the influence of various diagnostic methods for SIBO and MASLD on the results. Specifically, we compared breath tests and microbiota cultures, which are commonly used to diagnose SIBO, to assess how these methods may affect prevalence rates. Furthermore, our study systematically analyzed several potential confounding factors, including race, BMI, gender, and age, to explore their potential impact on the prevalence of SIBO in the context of MASLD. Moreover, our study also stands out for incorporating updated research data and adopting the latest diagnostic criteria for both SIBO and MASLD. Compared to previous studies, our research not only offers a broader and more comprehensive analytical framework but also demonstrates innovation in research design and data processing techniques. As a result, our findings provide greater representativeness and offer broader applicability, enhancing their relevance to clinical practice and potential patient management strategies.

### 4.3 Limitations of the meta-analysis

This meta-analysis had certain limitations. Various diagnostic methods for SIBO and liver disease were included and may have contributed to differences among the studies. Notably, only four articles that diagnosed SIBO through small intestine fluid extraction were included, which may compromise the reliability of the results. Future studies should assess additional articles that employ small intestine extraction as a diagnostic method to enable a more comprehensive and reliable analysis of SIBO. Furthermore, indicators related to intestinal permeability were not assessed, suggesting a need for further exploration and integration. Current research on the impact of BMI on the relationship between SIBO and MASLD is limited, and the underlying mechanisms remain unclear. Therefore, further studies are needed to investigate the role of BMI in this relationship and to conduct a more comprehensive analysis of the factors that may influence this association. Such research is essential to refine and expand the existing theoretical framework.

## 5 Conclusion

This study investigated the association between SIBO and MASLD using systematic screening and meta-analysis. The initial screening included 7,400 articles, of which 14 met the final inclusion criteria. A significant correlation between SIBO and MASLD was observed, with a comprehensive odds ratio of 3.09 (95% CI 2.09–4.59, *I*^2^ = 66%, *p* < 0.0001). Subsequent analysis revealed significant variations in SIBO positivity rates by MASLD type, with increasing rates correlating with disease progression. Lactulose breath tests offer more consistent results across different patient groups, while duodenal fluid culture provides a more sensitive diagnostic approach for detecting SIBO. Heterogeneity was low among studies in which MASLD was diagnosed by liver biopsy or including intestinal permeability testing was conducted, further substantiating the link between SIBO and MASLD. Racial factors and whether a region is developed or developing are sources of inter-group heterogeneity.

## Data Availability

The datasets presented in this study can be found in online repositories. The names of the repository/repositories and accession number(s) can be found in the article/Supplementary material.
