# Supplementary material for: Small intestinal bacterial overgrowth and metabolic dysfunction-associated steatotic liver disease
Source: Front Nutr. 2024 Dec 17;11:1502151. doi: 10.3389/fnut.2024.1502151 (PMC11685094; doi:10.3389/fnut.2024.1502151)
Supplement: Supplementary file 1 [file Data_Sheet_1.pdf]

This study is a meta-analysis conducted in accordance with Preferred Reporting Items for Systematic Reviews and Meta-Analyses (PRISMA) guidelines. The study protocol is registered with the International Prospective Register of Systematic Reviews (PROSPERO ID: CRD42023427040; URL:https://www.crd.york.ac.uk/)

Appendix 1. Sample of search strategies (Embase session results 15 Jul 2024).

No.	Query	Results
#47	#15 AND #46	532
#46	#16 OR #17 OR #18 OR #19 OR #20 OR #21 OR #22 OR#23 OR #24 OR #25 OR #26 OR #27 OR #28 OR #29 OR #30 OR #31 OR #32 OR #33 OR #34 OR #35 OR #36 OR #37 OR #38 OR #39 OR #40 OR #41 OR #42 OR #43 OR #44 OR #45	394,850
#45	masld	1,330
#44	metabolic AND dysfunction AND associated AND steatotic AND liver AND disease	1,262
#43	mafld	2,573
#42	'metabolic associated fatty liver disease'/exp OR 'metabolic associated fatty liver disease' OR (metabolic AND associated AND fatty AND ('liver'/exp OR liver) AND ('disease'/exp OR disease))	27,808
#41	liver AND fibrosis	129,873
#40	fibrosis, AND liver	129,873
#39	cirrhosis, AND liver	246,849
#38	cirrhosis, AND hepatic	74,428
#37	hepatic AND cirrhosis	74,428
#36	periportal AND fibrosis	2,340
#35	dietary AND liver AND cirrhosis	2,894
#34	dietary AND cirrhosis	2,976
#33	cryptogenic AND liver AND cirrhosis	3,066
#32	cirrhosis, AND liver	246,849
#31	cirrhosis AND hepatis	353
#30	cirrhosis	255,339

#29	liver AND periportal AND fibrosis	2,287
#28	steatohepatitis, AND nonalcoholic	32,628
#27	steatohepatitis, AND nonalcoholic	0
#26	nonalcoholic AND steatohepatitides	0
#25	nonalcoholic AND steatohepatitis	32,628
#24	nonalcoholic AND fatty AND liverss	3,750
#23	nonalcoholic AND fatty AND liver	79,227
#22	livers, AND nonalcoholic AND fatty	3,750
#21	liver, AND nonalcoholic AND fatty	79,227
#20	fatty AND livers, AND nonalcoholic	3,750
#19	fatty AND liver, AND nonalcoholic	79,227
#18	nonalcoholic AND fatty AND liver AND disease	71,279
#17	nafld	48,335
#16	'non alcoholic fatty liver disease'/exp OR 'nonalcoholic fatty liver disease' OR (non AND ('alcoholic'/exp OR alcoholic) AND fatty AND ('liver'/exp OR liver) AND ('disease'/exp OR disease))	81,838
#15	#1 OR #2 OR #3 OR #4 OR #5 OR #6 OR #7 OR #8 OR #9 OR #10 OR #11 OR #12 OR #13 OR #14	5,838
#14	upper AND gut AND bacterial AND overgrowth	135
#13	small AND intestine AND bacterial AND overgrowth	3,309
#12	small AND intestinal AND bacterial AND overgrowth AND syndrome	1,665
#11	small AND intestinal AND bacterial AND 'overgrowth'	20
#10	small AND gut AND bacterial AND overgrowth	1,131
#9	enteric AND bacteria AND overgrowth	236

#8	enteral AND bacterial AND overgrowth	362
#7	contaminated AND small AND bowel AND syndrome	48
#6	small AND intestine AND bacterial AND overgrowth AND syndrome	1,353
#5	sibo AND syndrome	922
#4	small AND intestinal AND bacterial AND overgrowth	3,821
#3	small AND bowel AND bacterial AND overgrowth	2,398
#2	sibo	2,712
#1	'small intestinal bacterial overgrowth'/exp OR 'small intestinal bacterial overgrowth' OR (small AND intestinal AND bacterial AND ('overgrowth'/exp OR overgrowth))	3,821

8

Appendix 2. Sample of search strategies (cochrane results 15 Jul 2024).

ID	Search	Hits
#1	small intestinal bacterial overgrowth	261
#2	small bowel bacterial overgrowth	156
#3	sibo syndrome	79
#4	small intestine bacterial overgrowth syndrome	64
#5	contaminated small bowel syndrome	9
#6	enteral bacterial overgrowth	24
#7	enteric bacteria overgrowth	22
#8	small gut bacterial overgrowth	119
#9	small intestinal bacterial over-growth	5
#10	small intestinal bacterial overgrowth syndrome	117
#11	small intestine bacterial overgrowth	149
#12	upper gut bacterial overgrowth	33
#13	#1 or #2 or #3 or #4 or #5 or #6 or #7 or #8 or #9 or #10 or #11 or #13	356
#14	Non alcoholic Fatty Liver Disease	3565
#15	NAFLD	3017
#16	Nonalcoholic Fatty Liver Disease	4279
#17	Fatty Liver, Nonalcoholic	4629
#18	Fatty Livers, Nonalcoholic	24
#19	Liver, Nonalcoholic Fatty	4629
#20	Livers, Nonalcoholic Fatty	24

#21	Nonalcoholic Fatty Liver	4629
#22	Nonalcoholic Fatty Livers	24
#23	Nonalcoholic Steatohepatitis	2017
#24	Nonalcoholic Steatohepatitides	0
#25	Steatohepatitides, Nonalcoholic	0
#26	Steatohepatitis, Nonalcoholic	2017
#27	liver periportal fibrosis	57
#28	liver periportal fibrosis	57
#29	Cirrhosis	12201
#30	cirrhosis hepatis	9
#31	cirrhosis, liver	11261
#32	cryptogenic liver cirrhosis	63
#33	dietary cirrhosis	359
#34	dietary liver cirrhosis	340
#35	periportal fibrosis	58
#36	Hepatic Cirrhosis	3858
#37	Cirrhosis, Hepatic	3858
#38	Cirrhosis, Liver	11261
#39	Fibrosis, Liver	5390
#40	Liver Fibrosis	5390
#41	MASLD	44
#42	metabolic dysfunction associated steatotic liver disease	42

#43	MAFLD	98
#44	metabolic dysfunction associated fatty liver disease	180
#45	#14 or #15 or #16 or #17 or #18 or #19 or #20 or #21 or #22 or #23 or #24 or #25 or #26 or #27 or #28 or #29 or #30 or #31 or #32 or #33 or #34 or #35 or #36 or #37 or #38 or #39 or #40 or #41 or #42 or #43 or #44	18513
#46	#13 and #45	65

Date Run: 15/07/2024 13:01:17

12 Appendix 3. Sample of search strategies (Pubmed results 15 Jul 2024).

Search number	Query	Search Details	Results	Time
1	(("naflds"[All Fields] OR "non alcoholic fatty liver disease"[MeSH Terms] OR ("non alcoholic"[All Fields] AND "fatty"[All Fields] AND "liver"[All Fields] AND "disease"[All Fields]) OR "non alcoholic fatty liver disease"[All Fields] OR "nafld"[All Fields] OR "non-alcoholic fatty liver"[All Fields] OR ("non alcoholic fatty liver disease"[MeSH Terms] OR ("non alcoholic"[All Fields] AND "fatty"[All Fields] AND "liver"[All Fields] AND "disease"[All Fields]) OR "non alcoholic fatty liver disease"[All Fields] OR "nash"[All Fields]) OR ("liver cirrhosis"[MeSH Terms] OR ("liver"[All Fields] AND "cirrhosis"[All Fields]) OR "liver cirrhosis"[All Fields] OR ("liver"[All Fields] AND "fibrosis"[All Fields]) OR "liver fibrosis"[All Fields]) OR ("liver cirrhosis"[MeSH Terms] OR ("liver"[All Fields] AND "cirrhosis"[All Fields]) OR "liver cirrhosis"[All Fields] OR "cirrhosis"[All Fields] OR "fibrosis"[MeSH Terms] OR "fibrosis"[All Fields]) OR ("fatty liver"[MeSH Terms] OR ("fatty"[All Fields] AND "liver"[All Fields]) OR "fatty liver"[All Fields] OR "steatohepatitis"[All Fields]) OR "mafld"[All Fields] OR "metabolic dysfunction-associated fatty liver disease"[All Fields] OR "masld"[All Fields] OR (("metabolic"[All Fields] OR "metabolical"[All Fields] OR "metabolically"[All Fields] OR "metabolics"[All Fields] OR "metabolism"[MeSH Terms] OR "metabolism"[All Fields] OR	(("naflds"[All Fields] OR "non alcoholic fatty liver disease"[MeSH Terms] OR ("non alcoholic"[All Fields] AND "fatty"[All Fields] AND "liver"[All Fields] AND "disease"[All Fields]) OR "non alcoholic fatty liver disease"[All Fields] OR "nafld"[All Fields] OR "non-alcoholic fatty liver"[All Fields] OR ("non alcoholic fatty liver disease"[MeSH Terms] OR ("non alcoholic"[All Fields] AND "fatty"[All Fields] AND "liver"[All Fields] AND "disease"[All Fields]) OR "non alcoholic fatty liver disease"[All Fields] OR "nash"[All Fields]) OR ("liver cirrhosis"[MeSH Terms] OR ("liver"[All Fields] AND "cirrhosis"[All Fields]) OR "liver cirrhosis"[All Fields] OR ("liver"[All Fields] AND "fibrosis"[All Fields]) OR "liver fibrosis"[All Fields]) OR ("liver cirrhosis"[MeSH Terms] OR ("liver"[All Fields] AND "cirrhosis"[All Fields]) OR "liver cirrhosis"[All Fields] OR "cirrhosis"[All Fields] OR "fibrosis"[MeSH Terms] OR "fibrosis"[All Fields]) OR ("fatty liver"[MeSH Terms] OR ("fatty"[All Fields] AND "liver"[All Fields]) OR "fatty liver"[All Fields] OR "steatohepatitis"[All Fields]) OR "mafld"[All Fields] OR "metabolic dysfunction-associated fatty liver disease"[All Fields] OR "masld"[All Fields] OR (("metabolic"[All Fields] OR "metabolical"[All Fields] OR "metabolically"[All Fields] OR "metabolics"[All Fields] OR "metabolism"[MeSH Terms] OR "metabolism"[All Fields] OR "metabolisms"[All Fields] OR "metabolism"[MeSH Subheading] OR "metabolities"[All Fields] OR "metabolization"[All Fields] OR "metabolize"[All Fields] OR "metabolized"[All Fields] OR "metabolizer"[All Fields] OR "metabolizers"[All Fields] OR "metabolizes"[All Fields] OR "metabolizing"[All Fields]) AND ("dysfunctional"[All Fields] OR "dysfunctionals"[All Fields] OR "dysfunctioning"[All Fields] OR "dysfunctions"[All Fields] OR "physiopathology"[MeSH Subheading] OR "physiopathology"[All Fields] OR "dysfunction"[All Fields]) AND ("associate"[All Fields] OR "associated"[All Fields] OR "associates"[All Fields] OR "associating"[All Fields] OR "association"[MeSH Terms] OR "association"[All Fields] OR "associations"[All Fields]) AND "steatotic"[All Fields] AND ("liver diseases"[MeSH Terms] OR ("liver"[All Fields] AND "diseases"[All Fields]) OR "liver diseases"[All Fields] OR ("liver"[All Fields] AND "disease"[All Fields]) OR "liver disease"[All Fields]))) AND (("SIBO"[All Fields] OR (("intestine, small"[MeSH Terms] OR ("intestine"[All Fields] AND	6347	10:26:34

	"metabolisms"[All Fields] OR "metabolism"[MeSH Subheading] OR "metabolities"[All Fields] OR "metabolization"[All Fields] OR "metabolize"[All Fields] OR "metabolized"[All Fields] OR "metabolizer"[All Fields] OR "metabolizers"[All Fields] OR "metabolizes"[All Fields] OR "metabolizing"[All Fields]) AND ("dysfunctional"[All Fields] OR "dysfunctionals"[All Fields] OR "dysfunctioning"[All Fields] OR "dysfunctions"[All Fields] OR "physiopathology"[MeSH Subheading] OR "physiopathology"[All Fields] OR "dysfunction"[All Fields]) AND ("associate"[All Fields] OR "associated"[All Fields] OR "associates"[All Fields] OR "associating"[All Fields] OR "association"[MeSH Terms] OR "association"[All Fields] OR "associations"[All Fields]) AND "steatotic"[All Fields] AND ("liver diseases"[MeSH Terms] OR ("liver"[All Fields] AND "diseases"[All Fields]) OR "liver diseases"[All Fields] OR ("liver"[All Fields] AND "disease"[All Fields]) OR "liver disease"[All Fields]))) AND (("SIBO"[All Fields] OR (("intestine, small"[MeSH Terms] OR ("intestine"[All Fields] AND "small"[All Fields]) OR "small intestine"[All Fields] OR ("small"[All Fields] AND "intestinal"[All Fields]) OR "small intestinal"[All Fields]) AND ("bacterial"[All Fields] OR "bacterially"[All Fields] OR "bacterials"[All Fields]) AND ("overgrowth"[All Fields] OR "overgrowths"[All Fields])) OR (("breath"[All Fields] OR "breathe"[All Fields] OR "breathed"[All Fields] OR "breathes"[All Fields] OR "breathings"[All Fields] OR "breaths"[All Fields] OR "respiration"[MeSH Terms] OR "respiration"[All Fields] OR	"small"[All Fields]) OR "small intestine"[All Fields] OR ("small"[All Fields] AND "intestinal"[All Fields]) OR "small intestinal"[All Fields]) AND ("bacterial"[All Fields] OR "bacterially"[All Fields] OR "bacterials"[All Fields]) AND ("overgrowth"[All Fields] OR "overgrowths"[All Fields])) OR (("breath"[All Fields] OR "breathe"[All Fields] OR "breathed"[All Fields] OR "breathes"[All Fields] OR "breathings"[All Fields] OR "breaths"[All Fields] OR "respiration"[MeSH Terms] OR "respiration"[All Fields] OR "breathing"[All Fields]) AND "test*"[All Fields]) OR ("curr issues intest microbiol"[Journal] OR ("intestinal"[All Fields] AND "microbiology"[All Fields]) OR "intestinal microbiology"[All Fields])) AND ("naflds"[All Fields] OR "non alcoholic fatty liver disease"[MeSH Terms] OR ("non alcoholic"[All Fields] AND "fatty"[All Fields] AND "liver"[All Fields] AND "disease"[All Fields]) OR "non alcoholic fatty liver disease"[All Fields] OR "nafld"[All Fields] OR "non-alcoholic fatty liver"[All Fields] OR ("non alcoholic fatty liver disease"[MeSH Terms] OR ("non alcoholic"[All Fields] AND "fatty"[All Fields] AND "liver"[All Fields] AND "disease"[All Fields]) OR "non alcoholic fatty liver disease"[All Fields] OR "nash"[All Fields]) OR ("liver cirrhosis"[MeSH Terms] OR ("liver"[All Fields] AND "cirrhosis"[All Fields]) OR "liver cirrhosis"[All Fields] OR ("liver"[All Fields] AND "fibrosis"[All Fields]) OR "liver fibrosis"[All Fields]) OR ("liver cirrhosis"[MeSH Terms] OR ("liver"[All Fields] AND "cirrhosis"[All Fields]) OR "liver cirrhosis"[All Fields] OR "cirrhosis"[All Fields] OR "fibrosis"[MeSH Terms] OR "fibrosis"[All Fields]) OR ("fatty liver"[MeSH Terms] OR ("fatty"[All Fields] AND "liver"[All Fields]) OR "fatty liver"[All Fields] OR "steatohepatitis"[All Fields]) OR "mafld"[All Fields] OR "metabolic dysfunction-associated fatty liver disease"[All Fields]))) OR "masld"[All Fields]

"breathing"[All Fields]) AND "test*"[All Fields]) OR ("curr issues intest microbiol"[Journal] OR ("intestinal"[All Fields] AND "microbiology"[All Fields]) OR "intestinal microbiology"[All Fields])) AND ("naflds"[All Fields] OR "non alcoholic fatty liver disease"[MeSH Terms] OR ("non alcoholic"[All Fields] AND "fatty"[All Fields] AND "liver"[All Fields] AND "disease"[All Fields]) OR "non alcoholic fatty liver disease"[All Fields] OR "nafld"[All Fields] OR "non-alcoholic fatty liver"[All Fields] OR ("non alcoholic fatty liver disease"[MeSH Terms] OR ("non alcoholic"[All Fields] AND "fatty"[All Fields] AND "liver"[All Fields] AND "disease"[All Fields]) OR "non alcoholic fatty liver disease"[All Fields] OR "nash"[All Fields]) OR ("liver cirrhosis"[MeSH Terms] OR ("liver"[All Fields] AND "cirrhosis"[All Fields]) OR "liver cirrhosis"[All Fields] OR ("liver"[All Fields] AND "fibrosis"[All Fields]) OR "liver fibrosis"[All Fields]) OR ("liver cirrhosis"[MeSH Terms] OR ("liver"[All Fields] AND "cirrhosis"[All Fields]) OR "liver cirrhosis"[All Fields] OR "cirrhosis"[All Fields] OR "fibrosis"[MeSH Terms] OR "fibrosis"[All Fields]) OR ("fatty liver"[MeSH Terms] OR ("fatty"[All Fields] AND "liver"[All Fields]) OR "fatty liver"[All Fields] OR "steatohepatitis"[All Fields]) OR "mafld"[All Fields] OR "metabolic dysfunction-associated fatty liver disease"[All Fields]))) OR (masld)

Appendix 4. Sample of search strategies (Ovid results 15 Jul 2024).

1	small intestinal bacterial overgrowth.af.	1187
2	small bowel bacterial overgrowth.af.	292
3	sibo syndrome.af.	5
4	small intestine bacterial overgrowth syndrome.af.	3
5	contaminated small bowel syndrome.af.	23
6	enteral bacterial overgrowth.af.	1
7	enteric bacteria overgrowth.af.	0
8	small gut bacterial overgrowth.af.	0
9	small intestinal bacterial over-growth.af.	6
10	small intestinal bacterial overgrowth syndrome.af.	37
11	small intestine bacterial overgrowth.af.	121
12	small intestine bacterial overgrowth.af.	121
13	upper gut bacterial overgrowth.af.	1
14	1 or 2 or 3 or 4 or 5 or 6 or 7 or 8 or 9 or 10 or 11 or 12 or 13	1578
15	Non alcoholic Fatty Liver Disease.af.	34809
16	Fatty Liver, Nonalcoholic.af.	31
17	Fatty Livers, Nonalcoholic.af.	0
18	Liver, Nonalcoholic Fatty.af.	15
19	Fatty Livers, Nonalcoholic.af.	0
20	Liver, Nonalcoholic Fatty.af.	15

21	Livers, Nonalcoholic Fatty.af.	0
22	Nonalcoholic Fatty Liver.af.	18051
23	Nonalcoholic Fatty Livers.af.	12
24	Nonalcoholic Steatohepatitis.af.	8463
25	Steatohepatitides, Nonalcoholic.af.	0
26	Nonalcoholic Steatohepatitides.af.	0
27	Steatohepatitis, Nonalcoholic.af.	29
28	liver periportal fibrosis.af.	6
29	liver periportal fibrosis.af.	6
30	Cirrhosis.af.	157195
31	cirrhosis hepatis.af.	27
32	cirrhosis, liver.af.	944
33	cryptogenic liver cirrhosis.af.	95
34	dietary cirrhosis.af.	37
35	dietary liver cirrhosis.af.	1
36	periportal fibrosis.af.	617
37	Hepatic Cirrhosis.af.	3962
38	Cirrhosis, Hepatic.af.	303
39	Cirrhosis, Liver.af.	944
40	Fibrosis, Liver.af.	651
41	Liver Fibrosis.af.	24700
42	mafld.af.	1953

43	metabolic dysfunction-associated fatty liver disease.af.	1040
44	masld.af.	1215
45	metabolic dysfunction associated steatotic liver disease. af.	0
46	15 or 16 or 17 or 18 or 19 or 20 or 21 or 22 or 23 or 24 or 25 or 26 or 27 or 28 or 29 or 30 or 31 or 32 or 33 or 34 or 35 or 36 or 37 or 38 or 39 or 40 or 41 or 42 or 43 or 44 or 45	197280
47	14 and 46	156

Ovid MEDLINE(R) and Epub Ahead of Print, In-Process, In-Data-Review & Other Non-Indexed Citations, Daily and Versions <1946 to July 12, 2024>

16

Appendix 5. Sample of search strategies (WOS results 15 Jul 2024).

Entitlements	#	Search Query	Database	Results	Date Run
- WOS.IC: 1993 to 2024<br>- WOS.CCR: 1985 to 2024<br>- WOS.SCI: 1996 to 2024<br>- WOS.AHCI: 1996 to 2024<br>- WOS.ESCI: 2019 to 2024<br>- WOS.ISTP: 2002 to 2024<br>- WOS.SSCI: 1996 to 2024<br>- WOS.ISSHP: 2002 to 2024	1	TS=(small bowel bacterial overgrowth)) OR TS=(small intestinal bacterial overgrowth)) OR TS=(sibo syndrome)) OR TS=(small intestine bacterial overgrowth syndrome)) OR TS=(contaminated small bowel syndrome)) OR TS=(enteral bacterial overgrowth)) OR TS=(enteric bacteria overgrowth)) OR TS=(small gut bacterial overgrowth)) OR TS=(small intestinal bacterial over-growth)) OR TS=(small intestinal bacterial overgrowth syndrome)) OR TS=(small intestine bacterial overgrowth)) OR TS=(small intestine bacterial overgrowth)) OR TS=(upper gut bacterial overgrowth)	All Databases	50	Tue Jul 16 2024 00:27:23 GMT+0800 (China standard time)
- WOS.IC: 1993 to 2024<br>- WOS.CCR: 1985 to 2024<br>- WOS.SCI: 1996 to 2024<br>- WOS.AHCI: 1996 to 2024<br>- WOS.ESCI: 2019 to 2024<br>- WOS.ISTP: 2002 to 2024<br>- WOS.SSCI: 1996 to 2024<br>- WOS.ISSHP: 2002 to 2024	2	#12 AND #14	All Databases	50	Tue Jul 16 2024 00:27:23 GMT+0800 (China standard time)

- WOS.IC: 1993 to 2024<br><br>- WOS.CCR: 1985 to 2024<br><br>- WOS.SCI: 1996 to 2024<br><br>- WOS.AHCI: 1996 to 2024<br><br>- WOS.ESCI: 2019 to 2024<br><br>- WOS.ISTP: 2002 to 2024<br><br>- WOS.SSCI: 1996 to 2024<br><br>- WOS.ISSHP: 2002 to 2024	3	TS=(Non alcoholic Fatty Liver Disease) OR TS=(NAFLD)) OR TS=(Fatty Liver, Nonalcoholic)) OR TS=(Fatty Livers, Nonalcoholic)) OR TS=(Liver, Nonalcoholic Fatty)) OR TS=(Livers, Nonalcoholic Fatty)) OR TS=(Nonalcoholic Fatty Liver)) OR TS=(Nonalcoholic Fatty Livers)) OR TS=(Nonalcoholic Steatohepatitis)) OR TS=(Nonalcoholic Steatohepatitides)) OR TS=(Steatohepatitides, Nonalcoholic)) OR TS=(Steatohepatitis, Nonalcoholic)) OR TS=(liver periportal fibrosis)) OR TS=(liver periportal fibrosis)) OR TS=(Cirrhosis)) OR TS=(cirrhosis hepatis)) OR TS=(cirrhosis, liver)) OR TS=(cryptogenic liver cirrhosis)) OR TS=(dietary cirrhosis)) OR TS=(dietary liver cirrhosis)) OR TS=(periportal fibrosis)) OR TS=(Hepatic Cirrhosis)) OR TS=(Cirrhosis, Hepatic)) OR TS=(Cirrhosis, Liver)) OR TS=(Fibrosis, Liver)) OR TS=(Liver Fibrosis)) OR TS=(mafld)) OR TS=(metabolic dysfunction associated fatty liver disease)) OR TS=(metabolic dysfunction-associated fatty liver disease)) OR TS=(masld)) OR TS=(metabolic dysfunction associated steatotic liver disease)	All Databases	195997	Tue Jul 16 2024 00:27:43 GMT+0800 (China standard time)
- WOS.IC: 1993 to 2024<br><br>- WOS.CCR: 1985 to 2024<br><br>- WOS.SCI: 1996 to 2024<br><br>- WOS.AHCI: 1996 to 2024<br><br>- WOS.ESCI: 2019 to 2024<br><br>- WOS.ISTP: 2002 to 2024<br><br>- WOS.SSCI: 1996 to 2024<br><br>- WOS.ISSHP: 2002 to 2024	4	#17 AND #15	All Databases	300	Tue Jul 16 2024 00:27:59 GMT+0800 (China standard time)
